# Dysregulation of miRNAs in Sicilian Patients with Autism Spectrum Disorder

**DOI:** 10.3390/biomedicines14010217

**Published:** 2026-01-19

**Authors:** Michele Salemi, Francesca A. Schillaci, Maria Grazia Salluzzo, Giuseppe Lanza, Mariagrazia Figura, Donatella Greco, Pietro Schinocca, Giovanna Marchese, Angela Cordella, Raffaele Ferri, Corrado Romano

**Affiliations:** 1 Oasi Research Institute-IRCCS, 94018 Troina, Italy; fran7.sch@gmail.com (F.A.S.); msalluzzo@oasi.en.it (M.G.S.); glanza@oasi.en.it (G.L.); mfigura@oasi.en.it (M.F.); dgreco@oasi.en.it (D.G.); pschinocca@oasi.en.it (P.S.); rferri@oasi.en.it (R.F.); cromano@oasi.en.it (C.R.); 2 Department of Surgery and Medical-Surgical Specialties, University of Catania, 95123 Catania, Italy; 3 Genomix4Life S.r.l., 84081 Baronissi, Italy; giovanna.marchese@genomix4life.com (G.M.); angela.cordella@genomix4life.com (A.C.); 4 Genome Research Center for Health-CRGS, 84081 Baronissi, Italy; 5 Department of Biomedical and Biotechnological Sciences, University of Catania, 95123 Catania, Italy

**Keywords:** autism spectrum disorder, microRNA, small RNA sequencing, inflammation, autoimmunity, cardiomyopathy

## Abstract

**Background:** Autism spectrum disorder (ASD) is a highly prevalent neurodevelopmental condition influenced by both genetic and non-genetic factors, although the underlying pathomechanisms remain unclear. We systematically analyzed microRNA (miRNA) expression and associated functional pathways in ASD to evaluate their potential as prenatal/postnatal, diagnostic, and prognostic biomarkers. **Methods:** Peripheral blood mononuclear cells from 12 Sicilian patients with ASD (eight with normal cognitive function) and 15 healthy controls were analyzed using small RNA sequencing. Differential expression analysis was performed with DESeq2 (|fold change| ≥ 1.5; adjusted *p* ≤ 0.05). Functional enrichment and network analyses were conducted using Ingenuity Pathway Analysis, focusing on Diseases and Biofunctions. **Results:** 998 miRNAs were differentially expressed in ASD, 424 upregulated and 553 downregulated. Enriched pathways were primarily associated with psychological and neurological disorders. Network analysis highlighted three principal interaction clusters related to inflammation, cell survival and mechanotransduction, synaptic plasticity, and neuronal excitability. Four miRNAs (miR-296-3p, miR-27a, miR-146a-5p, and miR-29b-3p) emerged as key regulatory candidates. **Conclusions:** The marked divergence in miRNA expression between ASD and controls suggests distinct regulatory patterns, thus reinforcing the central involvement of inflammatory, autoimmune, and infectious mechanisms in ASD, mediated by miRNAs regulating S100 family genes, neuronal migration, and synaptic communication. However, rather than defining a predictive biomarker panel, this study identified candidate miRNAs and regulatory networks that may be relevant to ASD pathophysiology. As such, further validation in appropriately powered cohorts with predictive modeling frameworks are warranted before any biomarker or diagnostic implications can be inferred.

## 1. Introduction

Autism spectrum disorder (ASD) is considered a broad neurodevelopmental disorder and a highly heterogeneous condition with a substantial hereditary component (40–90%). The term ASD encompasses autism, Asperger’s disorder, and pervasive developmental disorder not otherwise specified [1,2,3]. Historically, the diagnostic conceptualization of autism has undergone substantial revision. Earlier classifications, including DSM-IV-TR [4] and ICD-10 [5], distinguished between autism, Asperger’s disorder, and pervasive developmental disorder not otherwise specified, based on impairments across three core domains: social interaction, communication, and restricted or repetitive behaviors. With the introduction of DSM-5 [6], these categories were unified under the umbrella term autism spectrum disorder, and the diagnostic domains were consolidated into two core dimensions: deficits in social communication and interaction, and restricted, repetitive patterns of behavior, interests, or activities. This dimensional approach, subsequently adopted in ICD-11 [7], reflects the continuous and heterogeneous nature of ASD and aims to improve diagnostic consistency across clinical and research settings. 

Individuals with ASD frequently present with a broad range of neurodevelopmental, psychiatric, and medical comorbidities, which substantially contribute to the marked clinical heterogeneity of the disorder [1,8]. Commonly reported comorbid conditions include intellectual disability, epilepsy, sleep disorders, attention-deficit/hyperactivity disorder (ADHD), motor and sensory dysfunctions, as well as mood and anxiety disorders [1,8,9]. In addition, growing evidence supports the involvement of immune, inflammatory, and systemic alterations in a subset of individuals with ASD, suggesting that comorbidities may reflect shared underlying biological mechanisms rather than independent conditions [2,10,11]. The type, severity, and combination of these comorbidities vary widely across individuals and may influence both clinical trajectories and molecular profiles, highlighting the need for integrated clinical and biological approaches in ASD research [2,12]. 

The pathogenic mechanisms underlying ASD remain unknown, although the etiology is believed to be highly heterogeneous, involving both genetic and non-genetic factors. However, the genetic component does not play a significant causative role in ASD, since only 1–2% of ASD cases can be defined as highly genetic [1]. ASD is indeed a multifactorial condition, driven by genetic influences but strongly affected by prenatal and perinatal environmental factors such as maternal lifestyle, advanced maternal age, obesity, infections, gestational diabetes, exposure to valproic acid (VPA), assisted reproductive techniques, and short interpregnancy interval. It remains unclear whether the resulting interactions, leading to inflammatory damage, oxidative stress, hypoxia, and ischemia, are causal or coincidental [2,10,13,14].

From a genetic standpoint, several genes have been implicated, including *GPR37*, *PTPRZ1*, *CDH9*, *PTN*, *CASP2*, *GRM8*, *TAS2R1*, *CHD8*, *SCN2A*, and *SEMA5A*, which, together with prenatal and postnatal environmental factors, contribute to non-syndromic ASD [1,8,15,16]. By contrast, syndromic ASD is associated with chromosomal abnormalities or monogenic alterations (e.g., Rett syndrome, fragile X syndrome, and MECP2 duplication syndrome) [8]. To date, the marked heterogeneity of ASD means that its pathophysiology and a single, effective treatment remain elusive, hindering research strategies and the development of new therapeutic targets [11]. Voineagu et al. (2011) [17] conducted a transcriptomic study of postmortem brain tissue from individuals with ASD and healthy controls, revealing that many of the molecular abnormalities were related to transcriptional dysregulation and altered cortical splicing patterns, particularly those associated with A2BP1/FOX1 expression levels. Using RNA-seq analysis of the cerebral cortex in three neuropsychiatric disorders (ASD, schizophrenia, and bipolar disorder), Gandal et al. (2018) [18] demonstrated that alterations at the transcript isoform level are more strongly associated with disease-related brain changes. Their study was specifically designed to link putative causal factors of these disorders to molecular perturbations in the brain.

Recent studies suggest that ASD should not be viewed as a single disease but rather as a constellation of symptoms involving distinct gene networks regulated by multiple post-transcriptional mechanisms, including microRNAs (miRNAs) [19,20]. Understanding how genetic variants and/or their regulation, alone or in combination with environmental factors, contribute to ASD is complex, as these interactions may operate dynamically and vary across developmental stages [12]. Within this complex framework, the role of miRNAs in examining gene expression and functional pathways relevant to ASD, and in assessing their potential as prenatal/postnatal, diagnostic, and prognostic biomarkers, has become increasingly prominent. However, it is important to note that PBMC-derived miRNA expression profiles have been extensively investigated across a wide range of psychiatric, neurodevelopmental, and immune-mediated conditions, including schizophrenia, major depressive disorder, anxiety disorders, post-traumatic stress disorder, and systemic inflammatory diseases [21,22]. In most of these contexts, reported miRNA expression changes in PBMCs were typically modest in magnitude and often inconsistent across studies unless major biological and technical confounders, such as immune activation status, cytokine levels, or immune cell compositions, were explicitly modeled [23,24]. This body of literature underscores the sensitivity of PBMC miRNA profiling to inflammatory and compositional effects and highlights the need for cautious interpretation when broad miRNA dysregulation is observed.

miRNAs are small RNA molecules of no more than 25 nucleotides that regulate approximately two-thirds of messenger RNAs (mRNAs) through mRNA–miRNA interactions (for details on their function and structure, please see) [19,25]. A single miRNA can regulate hundreds of mRNAs and influence the expression of numerous genes. They play critical roles in the development and functioning of the central nervous system (CNS), contributing to neurogenesis, synaptogenesis, and neuronal migration. miRNAs circulate extracellularly and can cross the blood–brain barrier (BBB), regulating gene expression even in distant tissues. Accordingly, although the primary pathological target of ASD is the CNS, dysregulated miRNAs have also been detected in various peripheral tissues, including lymphoblasts, blood, saliva, and olfactory precursor cells, potentially explaining the comorbidities observed in some individuals [19,25].

The first studies on miRNA dysregulation in ASD date back to 2008, when Abu-Elneel et al. compared miRNA expression in post-mortem cerebellar cortical tissue from individuals with ASD and healthy controls, identifying several significantly dysregulated miRNAs. Their targets included well-established autism-related genes such as *NRXN1* and *SHANK3*, both essential to ASD phenotype development [26]. Subsequently, Ghahramani Seno et al. (2011) investigated transcriptional alterations and molecular networks in ASD, identifying dysregulated genes and miRNAs, including *HEY1*, *SOX9*, miR-486, and miR-181b, all of which participate in CNS development and function [27]. 

Most recently, Liu et al. (2025) [28] and Zheng et al. (2025) [29] explored VPA, one of the environmental factors implicated in this multifactorial disorder, in the context of environmental epigenetics, in which exosomes mediate epigenetic communication. Liu et al. found that prenatal VPA exposure reduced most exosomal miRNAs in the amygdala of newborns, particularly exosomal miR-215-5p, affecting the NEAT1/MAPK1/p-CRMP2 pathway and ultimately resulting in abnormal synaptic development and impaired social behavior. Zheng et al. analyzed plasma exosomal miRNA profiles in rats prenatally exposed to VPA versus controls. miR-30b-5p was significantly reduced in plasma exosomes and brain tissue, while inflammatory mediators (EGFR, p-p38/p38, CaMKII) were elevated in the brain. Restoration of miR-30b-5p attenuated neuroinflammation, suggesting a translational therapeutic potential for ASD [28,29]. Several studies on autism have used PBMCs. Such as Gevezova et al. (2021) who developed a study to evaluate changes in cellular bioenergetics and metabolism following mitochondrial dysfunction [30]. Jyonouchi et al. (2017) developed a study in which they analyzed both cytokine production and miRNA expression in ASD and CTRL subject groups [31]. Atwan et al. (2020) evaluated the gene expression of *BCL-2*, *IL-6*, miR-16, miR-181b-5p and miR-23a-3p, finding a difference between ASD and CTRL, as well as a lack of difference between sexes [32]. 

Based on these considerations, in this study, we performed a comprehensive and systematic analysis of miRNAs, followed by an in-depth functional characterization, to assess their potential dysregulation in patients with ASD compared to healthy controls (CTRL) and to elucidate the complex regulatory roles of miRNAs in modulating gene networks involved in ASD onset and progression. We hypothesized that patients with ASD would display a distinct and reproducible miRNA expression profile compared with CTRL, reflecting underlying neuroinflammatory and neurodevelopmental alterations, and that specific dysregulated miRNAs would be functionally linked to molecular pathways central to ASD pathogenesis and clinical manifestations.

## 2. Materials and Methods

### 2.1. Participants

The study enrolled 12 Sicilian patients diagnosed with ASD (10 males and 2 females; median age ± standard deviation: 17.58 ± 12.88 years) and 15 CTRL individuals (12 males and 3 females; median age ± standard deviation: 19.15 ± 12.07 years). All participants were recruited from the Oasi Research Institute–IRCCS in Troina (Italy) and were of Caucasian ancestry and Sicilian origin to minimize environmental variability. ASD diagnoses were established by a child neuropsychiatrist and a pediatric neurologist in accordance with the DSM-5 diagnostic criteria [6]. Diagnostic confirmation was obtained through neuropsychological and psychopathological assessments conducted by trained developmental psychologists. The Autism Diagnostic Observation Schedule–Generic (ADOS-G) or its revised version (ADOS-2) was also administered [33]. 

All participants, except for four ASD patients, had normal cognitive functioning. Two patients presented with moderate intellectual disability, and two with borderline intellectual disability. Language impairment was observed in four ASD children, developmental coordination disorder in two, ADHD in two (without severe disruptive behavior or other clinically relevant manifestations), and motor and vocal tic disorder in one. Electroencephalography findings were normal in all patients except for two: one exhibited clear epileptiform discharge and one showed sporadic non-specific alterations. The ASD cohort was selected to exclude individuals with comorbid neuropsychiatric disorders or ongoing pharmacological treatments. CTRL participants were medication-free, had no history of neurological or psychiatric disorders, and showed a completely normal neurological examination, with no evidence of underlying pathology.

Informed consent was obtained from all participants and their legal guardians. The study was conducted in accordance with the Declaration of Helsinki (1964) and its subsequent amendments. The study protocol was approved by the Ethics Committee of the Oasi-IRCCS Research Institute in Troina (Italy) on 10 April 2025 (approval code: CEL-IRCCS OASI/10-04-2025/01).

### 2.2. RNA Extraction 

Total RNA was isolated from peripheral blood mononuclear cells using TRIzol reagent (Invitrogen Life Technologies, Carlsbad, CA, USA) following the manufacturer’s instructions. RNA concentration and purity were measured with the NanoDropOne spectrophotometer (Thermo Fisher Scientific, Waltham, MA, USA), and RNA integrity was assessed with the Agilent TapeStation 4200 using the RNA ScreenTape Assay 2.7 (Agilent Technologies, Santa Clara, CA, USA). Extracted RNA was stored at −80 °C until further analysis.

### 2.3. RNA Sequencing and Data Analysis

Library preparation and sequencing were performed by Genomix4Life Srl (Baronissi, Italy). Indexed libraries were constructed from 250 ng of total RNA using the QIAseq miRNA Library Kit (QIAGEN, Hilden, Germany), following the manufacturer’s guidelines. Library quality and concentration were evaluated using the Agilent TapeStation 4200 (Agilent Technologies) and the Qubit fluorometer (Invitrogen-Thermo Fisher Scientific, Waltham, MA, USA), respectively. Equimolar pooling of indexed libraries was performed prior to cluster generation and sequencing on an Illumina NovaSeq 6000 platform (Illumina, Santa Clara, CA, USA) using a single-end 1 × 75 bp format. FASTQ files underwent quality control using FastQC. MicroRNA identification and quantification were carried out with sRNAbench [34] in library mode, using the Homo sapiens miRBase reference (release 22.1) [35]. Data normalization was performed in R using negative binomial generalized linear models, retaining genes expressed in at least 30% of samples.

Differential expression analysis between ASD and CTRL samples was performed using DESeq2 (version 1.50.1; Bioconductor 3.22) [36]. miRNAs with |fold change| ≥ 1.5 and adjusted *p*-value ≤ 0.05 were considered significantly differentially expressed. Data visualization included principal component analysis (PCA) and volcano plots generated with ggplot2 (version 4.4.3) [37], and heatmaps generated with ComplexHeatmap (version 2.20.0) [38]. Functional interpretation of differentially expressed miRNAs (DEmiRNAs) was conducted using Ingenuity Pathway Analysis (IPA) [39]. The IPA microRNA Target Filter was further used to link identified DEmiRNAs to experimentally validated or predicted mRNA targets (QIAGEN, 2000–2025). All data are publicly available in ArrayExpress under accession number E-MTAB-16206.

Differential expression analyses were conducted without explicit modeling of biological or technical covariates, such as age, sex, RNA extraction batch, PBMC cellular composition, or inflammatory status. Owing to the limited sample size and the absence of direct measurements for immune cell proportions and circulating cytokines, inclusion of these variables as covariates was not justified. Consequently, potential confounding effects could not be formally corrected, thus representing a potential limitation of the study.

## 3. Results

### 3.1. Small RNA Sequencing

**Figure 1:**
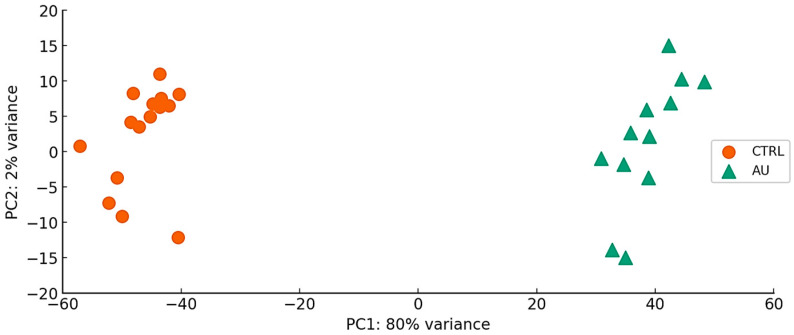
Principal Component Analysis (PCA) of ASD and CTRL samples showing distinct clustering of the two groups based on the first principal component (PC1) and second principal component (PC2), explaining 80% of the variance.

Small RNA expression profiling was performed using next-generation sequencing on peripheral blood samples from individuals with ASD and age-matched CTRL. Following adapter trimming and quality filtering, only high-quality reads were retained and subsequently aligned to the human reference genome. PCA revealed a distinct clustering pattern between ASD patients and controls, indicating global differences in small RNA expression profiles. However, this unsupervised separation should be interpreted descriptively and does not imply any diagnostic classification or predictive performance (Figure 1). As such, these analyses assessed internal consistency of the sequencing and analytical workflow, but did not control for potential biological or technical confounders.

Across all samples, a total of 1871 unique miRNAs were initially detected. After applying expression thresholds and quality filters, 1313 miRNAs were retained for downstream analysis. 

**Figure 2:**
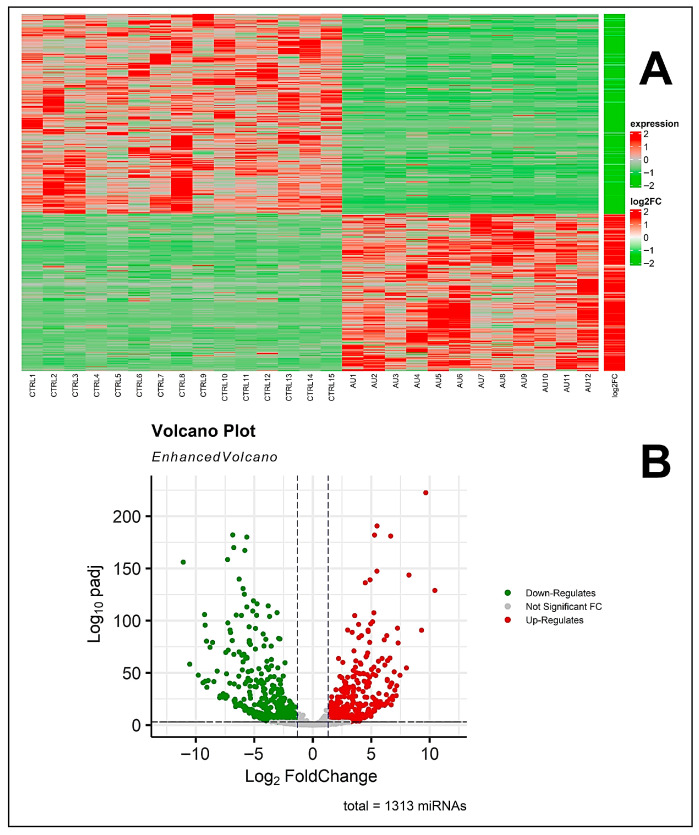
MicroRNAs (miRNAs) expression analysis. (**A**) Heatmap of the differentially expression profile of miRNAs found in ASD vs. CTRL. We reported in red the upregulated and in green the downregulated miRNAs, along with the bar of log2 (FoldChange). (**B**) Volcano plot displays the distribution of differentially expressed miRNAs.

Differential expression analysis between ASD and controls identified 998 significantly deregulated miRNAs (adjusted *p*-value ≤ 0.05), as illustrated in the heatmap (Figure 2A) and volcano plot (Figure 2B). Among these, 424 miRNAs were significantly upregulated (fold change ≥ 1.5), while 553 were significantly downregulated (fold change ≤ −1.5) (Table S1). It is relevant to note that the large number of differentially expressed miRNAs observed likely reflected a combination of biological variation and unmodeled technical or compositional factors inherent to PBMC-based profiling, rather than independent disease-specific effects for each miRNA, as further discussed below.

### 3.2. Analysis IPA

**Figure 3:**
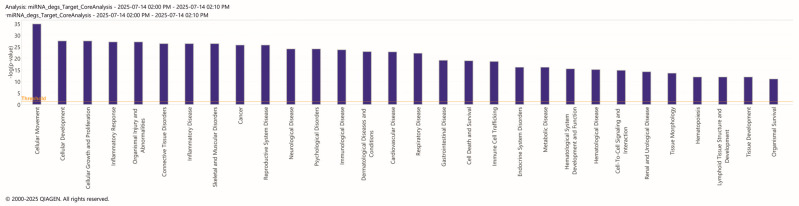
Bar Chart of the top 30 of Diseases and Biofunctions of Ingenuity Pathway Analysis (IPA) causally predicts the effects of molecular changes in the dataset on downstream biological processes (or “functions”) and diseases. Log(*p*-value) is shown on the *y*-axis.

To investigate the biological significance of these DEmiRNAs, we conducted a Core Analysis using IPA, that identified 63 statistically significant categories within the Diseases and Biofunctions domain, several of which were directly related to Psychological and Neurological Disease (Figure 3; Table S2).

Among the most notable pathways are Nervous System Development and Function, Cellular Growth and Proliferation, and Cell-to-Cell Signaling. These results suggest that small RNAs may modulate fundamental CSN processes, such as neurogenesis and synaptic connectivity, potentially contributing to the behavioral deficits observed in patients with ASD. Beyond strictly neurological pathways, there is also enrichment in pathways associated with behavioral and cognitive disorders, including behavior, psychological disorders, and developmental disorders. This suggests that small RNAs play a significant role in the regulation of behavioral and cognitive circuits, supporting the hypothesis that the dysregulation of specific miRNAs may contribute to the pathogenesis of ASD through combined effects on neuronal development and related neural processes.

### 3.3. Analysis of the Differentially Expressed miRNAs’ Target Genes

Using IPA, we performed a miRNA Target Filter analysis to explore the relationships between DEmiRNAs and their predicted or validated mRNA targets. This tool integrates experimentally confirmed and computationally predicted interactions from the QIAGEN Ingenuity Knowledge Base, enabling prioritization of target genes based on our dataset.

**Figure 4:**
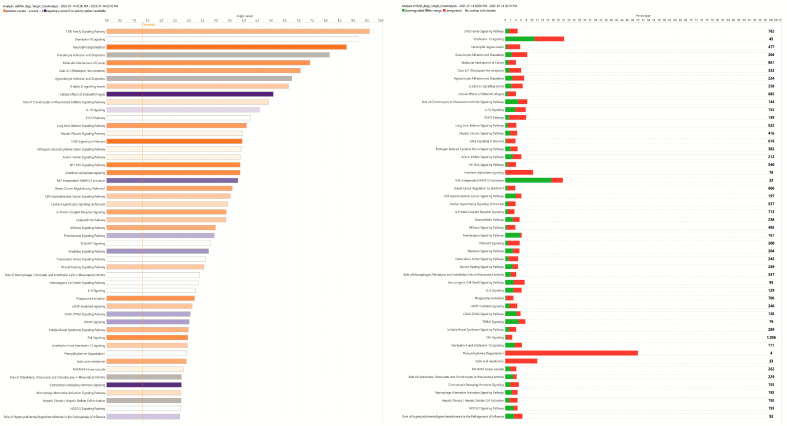
Horizontal Bar Charts of the top 50 of Canonical Pathway Ingenuity Pathway Analysis (IPA) on microRNA (miRNA) target genes. The pathway names are along the *y*-axis, whereas the *x*-axis displays the negative log of the *p*-value such that taller bars equate to increased significance. The bars are sorted so that the most significant pathways are on the top of the chart. The orange and blue-colored bars in the bar chart indicate predicted pathway activation or predicted inhibition, respectively, via another statistic: the z-score. The Number of Up- and Down-expressed genes involved in each enriched pathway are show in the right column. where the *x*-axis represents the percentage of molecules that are present in a specific Canonical Pathway, while those molecules are represented in the *y*-axis in the Vertical Stacked Bar Chart view.

Since RNA-seq analysis had previously been conducted on the same cohort of ASD and control samples (PIMD accession: E-MTAB-13871), we cross-referenced the 998 DEmiRNAs identified in this study with the differentially expressed genes detected in the RNA-seq dataset. To refine the results, we selected only those miRNA–mRNA interactions that: (i) were involved in at least one canonical pathway, and (ii) were supported by a Confidence Score classified as High-Prediction and/or Experimentally Observed according to IPA (Table S3). This filtering process yielded 2467 high-confidence miRNA–mRNA interactions, comprising 637 unique miRNAs and 256 target genes. Based on this refined interaction set, a second Core Analysis was conducted in IPA, which revealed 712 significantly enriched Canonical Pathways. Among these, the top 50 pathways were selected for detailed investigation (Table S4; Figure 4). Among the significantly enriched Canonical Pathways, prioritization was based on statistical significance and predicted pathway activity, with emphasis placed on pathways functionally relevant to the biological context of this study.

IPA Network analysis of differentially expressed miRNAs and their predicted targets identified multiple enriched interaction networks.

### 3.4. Networks

**Figure 5:**
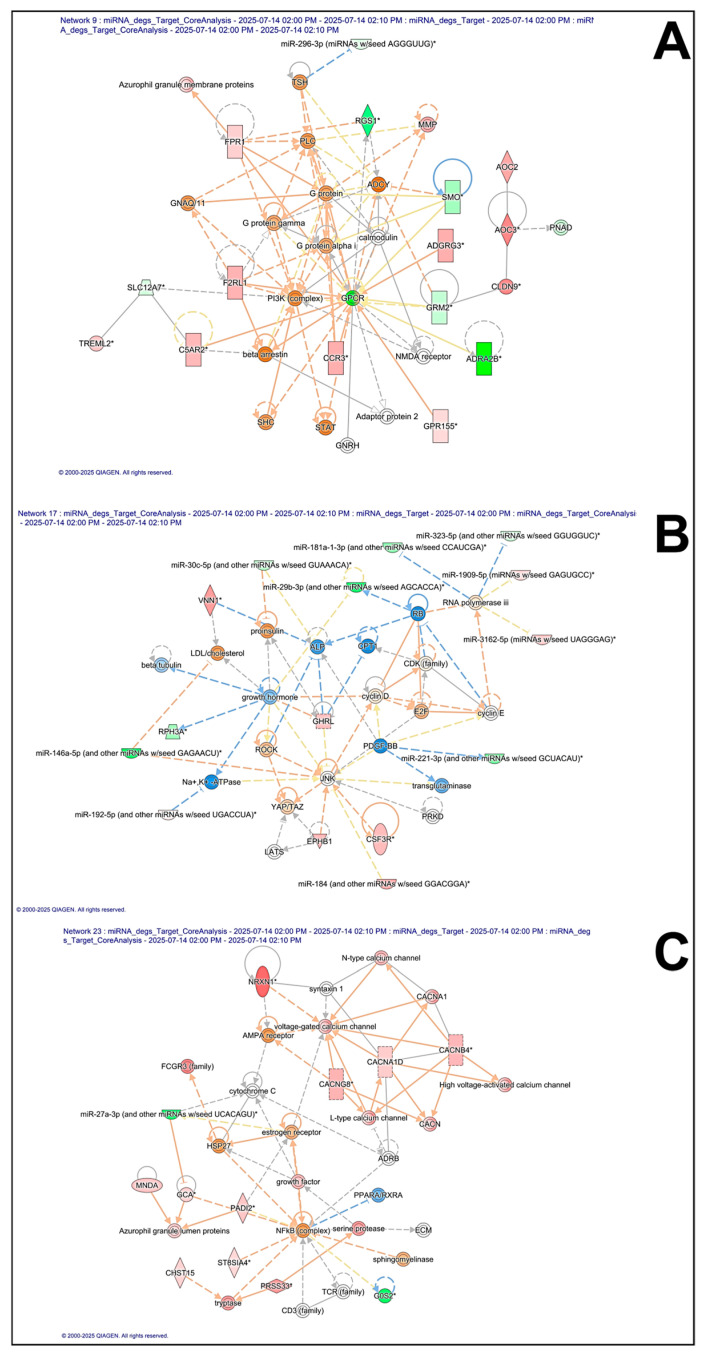
Ingenuity Pathway Analysis (IPA) networks (**A**) = network 9, (**B**) = network 17, (**C**) = network 23; Supplementary Table S5 associated with various diseases and functions. Molecule colors reflect expression values: red = upregulation, green = downregulation, gray = molecules present but below the user-defined cutoff, white = molecules added from the Ingenuity Knowledge Base. Lines indicate molecular interactions (solid = direct, dashed = indirect), and arrows represent the type and direction of the relationship. * = mature miRNA.

We focused on three networks (ID: 9, 17 and 23 Table S5, Figure 5) due to their high scores and biological relevance. Network 9 centers on GPCR-mediated signaling, PI3K/PLC pathways, chemotactic/immune mediators and encompasses miR-296-3p (log2FC = −1.65), indicating a possible neuro-immune interface. This pattern implies a reorganization of immune signaling networks rather than a simple increase or decrease in inflammatory tone, supporting the concept of immune dysregulation as a feature of ASD.

Network 23 comprises voltage-gated calcium channel subunits (*CACNA1D*, *CACNA1E*, *CACNB4*, and *CACNG8*), synaptic components (e.g., *NRXN1*, syntaxin 1) and miRNA regulators (miR-27a which is strongly downregulated log2FC = −5.97), suggesting potential alterations in synaptic transmission. In particular, voltage-gated calcium channel subunits are known to play a crucial role in neuronal excitability, neurotransmitter release, and synaptic plasticity, and their upregulation suggests a hyper-activation of calcium-dependent signaling pathways. This potentially leads to an imbalance in excitatory/inhibitory transmission (E/I imbalance), which is a hallmark of ASD neurobiology. NRXN1 (Neurexin 1) is a presynaptic cell adhesion molecule essential for synapse formation and specification through its interactions with neuroligins. 

As such, its overexpression may reflect a compensatory synaptic remodeling mechanism or a pathological enhancement of synaptic signaling, both consistent with previous ASD transcriptomic reports. Lastly, Network 17 contains factors linked to cell survival and mechanotransduction (YAP/TAZ, ROCK), as well as miRNAs involved in inflammatory regulation (miR-146a-5p (log2FC = −5.43), miR-221-3p (log2FC = −4.02) and miR-29b-3p (log2FC = −4.25), pointing to interplay between stress responses and neuronal signaling.

**Figure 6:**
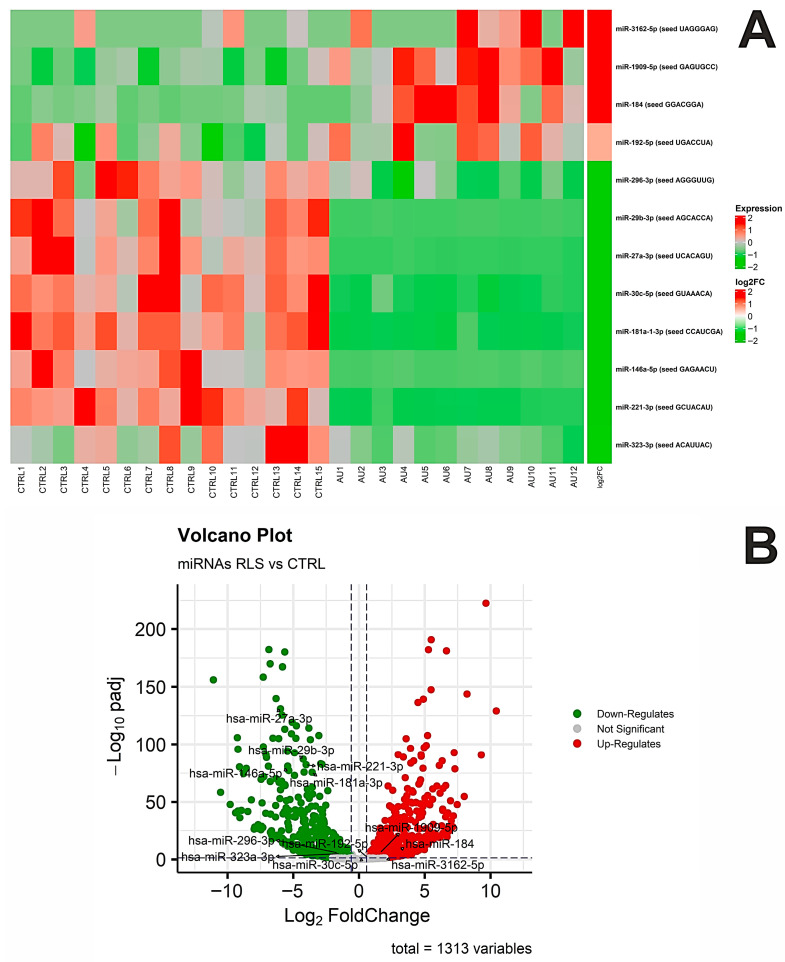
(**A**) Heatmap showing the differential expression profiles of miRNAs identified within the three networks in ASD versus CTRL samples. Upregulated miRNAs are shown in red, while downregulated miRNAs are shown in green, together with the corresponding log_2_(Fold Change) scale. (**B**) Volcano plot illustrating the distribution of the differentially expressed miRNAs identified in the three networks.

Of particular interest are the small RNAs identified within three networks (Figure 6), since they reinforce the hypothesis of a role for post-transcriptional regulation within the pathomechanisms of ASD. Namely, the analyzed networks revealed deregulated miRNAs that act as control nodes of genes involved in both synaptic function and immune response. This suggests that the observed alterations were not the result of isolated single genes, but rather of a coordinated reorganization of entire molecular circuits regulated by miRNAs. Overall, therefore, these findings highlight miRNA-mediated post-transcriptional control as a major regulatory layer contributing to the observed expression patterns. Indeed, miRNAs are known to fine-tune both synaptic and immune genes, and their dysregulation may bridge neuronal and immune alterations in ASD. Nevertheless, the miRNAs highlighted within these networks (miR-296-3p, miR-27a, miR-146a-5p, and miR-29b-3p) were selected based on network centrality and biological relevance rather than predictive performance. As such, these miRNAs do not constitute a diagnostic or predictive panel, and no supervised classification, feature selection, or model training was performed to assess their ability to discriminate ASD cases from controls.

## 4. Discussion

### 4.1. IPA

Before discussing the biological implications of the identified miRNA signatures, it is essential to acknowledge that the high number of differentially expressed miRNAs and the magnitude of some fold changes raise the possibility of non-biological contributions to the observed signal. PBMCs cells represent indeed a heterogeneous cellular population and differences in immune cell composition, platelet contamination, hemolysis, or low-abundance miRNA noise may have inflated apparent differential expression. Accordingly, the present results should be interpreted as reflecting global regulatory shifts rather than as evidence that hundreds of individual miRNAs acted as independent disease markers. Although several workflow-level robustness checks were performed, including PCA stability, flow-cell consistency, and concordance across analytical steps, these procedures address technical reliability rather than confounding. Also, as stated, no explicit modeling or correction was applied for potential covariates, such as sex, age, batch effects, PBMC subpopulation composition, or inflammatory status. As a result, the observed miRNA expression differences may reflect, at least in part, unmeasured biological or technical confounders rather than ASD-related regulatory mechanisms alone.

In this context, the present findings should not be interpreted as evidence that ASD is uniquely associated with stronger or more extensive PBMC miRNA dysregulation than other psychiatric or neurodevelopmental disorders. Rather, the observed breadth of differential expression may reflect enrichment of immune-related and inflammatory processes within the selected ASD cohort, combined with the inherent sensitivity of small RNA sequencing to cellular composition and immune activation state. Indeed, prior PBMC-based miRNA studies in schizophrenia, mood disorders, and anxiety disorders have demonstrated that robust or reproducible signatures often emerge only when inflammatory markers, cytokine levels, or immune cell proportions are explicitly incorporated into the analytical framework [23,24].

With these considerations in mind, the variance shown in Figure 1 (PCA) indicates that ASD and CTRL significantly differ. Overall, the ASD cases were relatively homogeneous from a cognitive standpoint; individuals with intellectual disability did not differ substantially from the rest of the cohort, nor were there meaningful differences related to sex. Although the preliminary nature of the study, this may allow to hypothesize that inflammatory, autoimmune, and infective mechanisms play a crucial role in ASD, emphasizing, in particular, the specific function of miRNAs that regulate genes in the S100 family. Another important finding observed was the pivotal role of regulatory mechanisms, via miRNa, which modulate neuronal migration and communication systems, such as synapses that occur during the development of the CNS, as well as the possible comorbidity between cardiomyopathies and ASD.

### 4.2. Diseases and Biofunctions of Ingenuity Pathway Analysis (IPA)

The bar chart in Figure 3, in which we have identified different statistically significant categories within the Diseases and Biofunctions domain, shows that the basic analysis performed with IPA identifies “Cell Movement” as the Biofunctions domain with the highest number of differentially expressed miRAs in ASD compared to CTRL. During embryonic development of vertebrates, cellular migration or movement by individual cells or cell clusters (collective migration) is crucial for forming tissues and organs [40].

For instance, gastrulation in early embryos requires mass movements of specific cell populations to form the three primary tissue layers. Migrating neural crest cells disperse in precise patterns throughout the body to give rise to sympathetic and sensory ganglia. Similarly, cancer metastasis can involve a conversion of cells from epithelial to invasive mesenchymal cells or the invasive protrusion of masses of tumor cells into normal tissues [41]. A neuropathology studies reveals that patients with ASD show phenotypes associated with cell migration, including changes in neuronal density and volume [42]. Furthermore, another study revealed multiregional dysregulation of neurogenesis, neuronal migration, and maturation, contributing to the heterogeneity of the clinical phenotype of ASD [43]. In a study by Stoner et al. (2014), focal areas of abnormal laminar cytoarchitecture and cortical disorganization of neurons, but not glia, were found in the prefrontal and temporal cortical tissue of most brain samples from children with ASD [44]. The fact that there was disorganization of neurons but not glia suggests an abnormality in mid-gestation.

Of note, some of the above-mentioned studies were performed on post-mortem brain histological material from subjects with ASD. The data obtained highlight the abnormal migration of brain neurons in subjects with ASD. On the other hand, our study on leukocytes, although preliminarily performed on a small sample of subjects with ASD, highlights the dysregulation of miRNAs that regulate cell movement. This suggest that the same mechanisms that activate neuronal movements may also be activated in the leukocytes in terms of miRNA expression. Therefore, the leukocyte model could be representative for studies of gene regulation in individuals with ASD.

A correlation between pathomechanisms and neuroimmune connections has been recognized, although hypothesized in dated studies, through some dysfunctions that can occur either in the prenatal or postnatal period [11,45,46,47]. In a descriptive study spanning almost a decade, the connection between the gut/brain axis and neuroinflammation was evaluated, linking them to the hypothetical pathophysiology of ASD. In particular, the authors considered that dysregulated immune cell traffic (Claudin (CLDN)-5 and -12, CLDN-3, tricellulin, MMP-9) on both the intestinal barrier and BBB was the basis for episodes of neuroinflammation [48]. ASD is a multifactorial disorder, and a possible link between the gut/brain/neuroinflammation might be the result of several factors, including genetic, autoimmune, environmental, prenatal, and postnatal factors. It has been found that cytokine alterations reflect the severity of patients’ symptoms. In particular, they detected elevated levels of IL-18 and adhesion factors (ICAM-1, MADCAM1), both systemically and centrally, and a possible role for the NLRP3 inflammasome, as its inhibition in vitro mimicked the immune phenotype of individuals with ASD [11,49]. Therefore, several studies have highlighted inflammatory impairment and the role of cell adhesion molecules, raising the question of whether there is a dysfunction in the modulation of neuroimmune signaling or in glial behavior, the main source of cytokines [50]. Our study highlighted several immune implications (Figure 3 and Figure 5, Table S5), which, together with the existing literature, may provide a pathophysiology contribution for ASD. Namely, these findings support a model in which ASD could arise from combined synaptic and immunoregulatory dysfunctions, possibly underpinned by epigenetic and miRNA-dependent mechanisms. This molecular profile is also consistent with current hypotheses that position ASD as a neurodevelopmental disorder with a strong immune-synaptic component.

Some studies have hypothesized that the etiology of ASD focuses on abnormal brain growth and dysregulation of connectivity between regions [51]. Subsequently, in their proteomic study, Fatemi et al. (2025) detected protein downregulation that impacted various mechanisms (including protein folding, aggrephagy, and anterograde/retrograde transport, endocytosis) and concluded that these dysfunctions were already present during the fetal period and continued into adulthood [52].

### 4.3. DEmiRNA

Regarding extra neurological manifestations, miR-296-3p is a miRNA that has been detected mainly in the context of cancer, but also in cardiomyopathies and in certain neurological syndromes [53]. To our knowledge, this is the first time this miRNA was observed in individuals with ASD. The miRNA has been identified in papillary thyroid carcinoma, whose malignancy has been hypothesized to be associated with high iodine levels and the circ_0004851/miR-296-3p/FGF11 axis [54]. In triple-negative breast cancer, it has been found that overexpression of miR-296-3p inhibits the Wnt/β-catenin signaling pathway by targeting SOX4 and exerting antitumor effects [55]. Other tumors involved are the colorectal cancer [56], the pancreatic ductal adenocarcinoma [57], and the laryngeal squamous cell carcinoma [58]. 

A study identified a correlation between the NLR Family, Pyrin Domain-Containing 3 gene (NLRP3), which is involved in inflammation, the incidence of acute myocardial infarction, and the expression level of miR-17-3p, miR-101-3p, miR-335-3p, and miR-296-3p [59]. In another study, however, under-expression of this miRNA correlated with high expression of the target VEGF-B, predicts the degree of coronary artery stenosis in patients with coronary heart disease) [60]. CRISPR/Cas9, a powerful and recent genetic editing technology, also targets miRNAs, which have been identified as regulators of the cardiovascular system [61]. Recently, Kaya et al. (2025) used a mouse model that emulates Tay-Sachs disease to further investigate the identification of miRNAs in order to identify therapeutic targets or develop targeted therapies [62]. The authors detected several miRNAs: miR-708-5p, miR-672-5p, miR-204-5p, miR-335-5p, and miR-296-3p were upregulated, while miR-10b-5p, miR-615-3p, miR-196a-5p, miR-214-5p, and miR-199a-5p were downregulated. Another study focused on the molecular similarities between Parkinson’s disease and cancer, as well as on genes related to differentially expressed disulfideptosis and miRNAs common to both conditions [63,64,65,66]. Schizophrenia, a very challenging neuropsychiatric disorder was the focus of a recent study by Sowmya Gunasekaran et al. They focused on the regulation of N-methyl-D-aspartate receptors, suggesting that the under-expression of the genes Glutamate Receptor, Ionotropic, N-Methyl-D-Aspartate, Subunit 2a (*GRIN2A*) and Glutamate Receptor, Ionotropic, N-Methyl-D-Aspartate, Subunit 2b (*GRIN2B*) genes was regulated by miRNAs (MiR-296-3p, miR-148b-5p, and miR-137) [67]. Therefore, miR-296-3p has been highlighted in various conditions spanning from neurodegenerative [63,67], cardiac [59,60,61], and neurodevelopmental disorders [62], thus hypothesizing that the gene(s) dysregulated by miRNA may exert different effects on the development of different diseases.

MIR27A is a microRNA which is implicated in various biological processes and diseases, including diseases caused by alterations in mitochondrial DNA [68,69]. This miRNA is involved in the regulation of the Nrf2 pathway, which is crucial for cellular defense, by binding to Nrf2 through multiple distinct sites when co-existing with other miRNAs [70]. Also, MIR27A has been studied for its association with the risk of coronary artery disease, thus highlighting its potential role in cardiovascular disorders [71]. Furthermore, MIR27A is suggested to have a regulatory role in both stem cell and adipocyte differentiation processes, and its over-expression has been observed to inhibit the migration of preadipocytes [72]. This miRNA also interacts with key transcription factors and signaling pathways by regulating Sp1 repressors involved in the expression of Vascular Endothelial Growth Factor (VEGF) and VEGFR1 [73]. Furthermore, a study by Baulina et al. (2016) highlights how various miRNAs, including miR-27a, are dysregulated in autoimmune mechanisms [74]. In their recent review, Liao and Li (2020) [75] summarized the existing publications on the genetic associations between voltage-gated calcium channels (VGCCs) and ASD. The most prominent among the VGCCs variants found in ASD were those falling within loci encoding the α subunits, CACNA1A, CACNA1B, CACNA1C, CACNA1D, CACNA1E, CACNA1F, CACNA1G, CACNA1H, and CACNA1I as well as those of their accessory subunits CACNB2, CACNA2D3, and CACNA2D4. Taken together, this supports the role of *VGCC* genes in the pathogenesis of ASD and the role of these genes targeting the miR-27a (Figure 5).

MIR221 is another important miRNA implicated in various cellular processes, including apoptosis and autophagy, as well as in the pathogenesis of multiple hyperlipidemia-related diseases and cancer [76,77,78,79]. Inhibition of MIR221 has been shown to increase apoptosis in transfected cells [76]. Moreover, MIR221 appears to be involved in the regulation of autophagic flux, as evidenced by the aggregation of P62 in cells transfected with MIR221 mimics [77]. In the context of atherosclerosis and vascular calcification, MIR221 is one among several miRNAs whose expression levels are altered [80]. Additionally, MIR221 has been identified as an onco-miRNA, that can promote cancer cell growth by targeting *PTEN* expression and activating oncogenic pathways such as PI3K/Akt/mTOR [79,81]. The expression level of MIR221 can be normalized to U6 RNA for analytical purposes [82] and its levels along with other clinical features can be used to predict patient outcomes in diseases such as the hyperlipidemia-related liver cancer associated with cholangiocarcinoma using statistical models [83].

MIR29B1 is a microRNA gene that encodes for the miR-29b-3p sequence, a member of the miR-29 family, which is implicated in various cellular processes and diseases [84]. In patients with Alzheimer’s disease (AD), MIR29B1 has been found to be downregulated, suggesting a potential role in the disease’s pathogenesis [85]. This downregulation may influence AD by targeting genes directly involved in its pathogenesis, such as *BACE1*, which deals with amyloid precursor protein processing. Additionally, MIR29B1’s downregulation might indirectly affect neuron survival, thus contributing to AD progression [85]. In other studies, MIR29B1 has been associated with various biological processes; for instance, in increasing milk yield in dairy cattle when co-expressed with MIR148A [86], and its upregulation was observed upon knockdown of LASP-1 correlating with reduced MMP9 transcript levels in MDA-MB-231S cells [87]. Furthermore, mutations in MIR29B1 have been linked to altered physiological responses, such as reduced urine volume and increased renal fibrosis under certain dietary conditions in rats [88]. Overall, this underscores its significance also beyond the neurodegenerative diseases.

Nevertheless, the present study lacks experimental validation of the sequencing-based findings: no orthogonal assays, such as quantitative PCR, synthetic spike-in controls, or independent replication cohorts, were employed to confirm differential miRNA expression. Consequently, the reported fold changes and significance levels should be interpreted cautiously and the identified miRNAs regarded as candidates for future targeted validation rather than confirmed biomarkers or a diagnostic panel.

Also, an important point of clarification concerns the interpretation of the identified miRNAs in relation to disease prediction. The present study did not propose a PBMC-based miRNA panel that is strongly predictive of ASD, nor did it estimate predictive power, sensitivity, specificity, or classification accuracy. While unsupervised analyses revealed group-level separation, no supervised learning, cross-validation, or independent test set was employed, precluding any inference regarding predictive performance. Accordingly, the miRNAs discussed in this work should be regarded as biologically prioritized candidates emerging from network-level analyses rather than as components of a clinically actionable signature. Future studies specifically designed for prediction, e.g., incorporating covariate modeling, feature selection, and external validation, are required to determine whether a reduced miRNA panel has discriminative utility in ASD.

### 4.4. Canonical Pathway Ingenuity Pathway Analysis (IPA) on microRNA (miRNA) Target-Genes

The horizontal bar chart of the 50 main canonical pathways derived from the IPA on miRNA target genes highlights how various miRNAs belong to specific pathways. The S100 protein family consists of 24 members functionally distributed into three main subgroups: (i) those that only exert intracellular regulatory effects; (ii) those with intracellular and extracellular functions; (iii) those which mainly exert extracellular regulatory effects. Within cells, S100 proteins are involved in aspects of regulation of proliferation, apoptosis, differentiation, energy metabolism, Ca^2+^ homeostasis, inflammation, and migration/invasion through interactions with a variety of target proteins, including enzymes, cytoskeletal subunits, receptors, transcription factors, and nucleic acids [89]. Thus, extracellular S100 proteins exert regulatory activities on monocytes/macrophages/microglia, neutrophils, lymphocytes, mast cells, articular chondrocytes, endothelial and vascular smooth muscle cells, neurons, astrocytes, Schwann cells, epithelial cells, myoblasts, and cardiomyocytes, thereby participating in innate and adaptive immune responses, cell migration and chemotaxis, tissue development and repair, and leukocyte and tumor cell invasion [89].

S100A9 is a major cytosolic protein in monocytes; the monocyte count is typically elevated in autism [90,91]. The independent association between S100A9 levels and Childhood Autism Rating Scale scores confirmed and expanded previous findings on the role of immune activation in the pathogenesis of ASD [91]. Neurological development and neuroarchitecture are fundamental in the pathogenesis of autism and astrocytes, along with other glial cells, are crucial in this context. In particular, S100B is expressed in these glial cells and, after brain injury, S100B levels increase in the serum of patients. In patients with ASD, it was found that in many cases S100 levels were elevated [92,93]. Astrocytes are the most abundant source of S100B in the human brain and may play a role in the pathogenesis of ASD. 

Taken together, these data suggest a role for inflammation and immune activation in the pathogenesis of ASD. In this scenario and in line with the existing literature [94], new insights supporting multisystem-related features in ASD might emerge, which would strengthen the link between autism and cardiomyopathy or other medical conditions, such as inflammatory bowel disease, infections, cerebral palsy, muscular dystrophy, and schizophrenia [95,96], although further evidence is needed to confirm this data. Notably, the study by Bilder et al. (2013) highlights that respiratory, cardiac, and epileptic events have been reported among the leading causes of death in ASD [97]. The cardiovascular reasons include myocarditis, arrhythmia, congestive heart failure, and congenital heart disease. These data from the literature seem to correlate, at least partially, with our preliminary data, which highlight approximately 500 dysregulated miRNAs that play a role in the mechanisms associated with hypertrophic cardiomyopathy.

Lastly, a recent review by Katoh et al. describes all the roles for focal adhesion kinase (FAK). FAK is located in focal adhesions, which are essential for cell–cell contact and communication, being also involved in transfer into and out of cells. In addition, this kinase is important for cell migration and angiogenesis, cell proliferation, and regulation of cell behavior [98]. ASD is still a cryptic disease, and a primary goal of research in this field is to decipher the molecular mechanisms of various neurodevelopmental conditions. FAK is part of the non-receptor tyrosine kinase family and plays a role in axonal guidance and neurodevelopmental signaling, being therefore a significant impact on neurodevelopmental disorders [99]. In this context, since cell migration is believed to play a role in the pathological processes leading to ASD, Pang et al. (2023) developed a mouse knockout for the Tetraspanin 7 (*TSPAN7*) gene, a model that induced an autistic phenotype, impairment of neuronal synapses, and downregulation of the integrin β1/FAK/SRC signaling pathway [100]. Consistently, Wei et al. (2011) demonstrated a reduction in FAK and other proteins important for neuronal migration, axonal branching, and synapse formation, as well as an IgG deficiency [101]. All these functions and mechanisms were found to be altered in our analyses from peripheral blood cells of ASD patients (Figure 3 and Figure 4, Table S5).

### 4.5. Limitations

Several limitations should be considered when interpreting the results of this study. First, the relatively small sample size may limit statistical power and the robustness of the findings. Second, although the observed male predominance reflects the well-known higher prevalence of autism in males and the tendency for ASD to be under-recognized in females [102,103], the resulting sex imbalance may restrict the generalizability of the results to the female autistic population. Accordingly, future studies including larger and more balanced cohorts of males and females will be necessary to further investigate sex-related differences in miRNA expression and to confirm or refute these preliminary findings.

With respect to environmental influences, the exclusive recruitment of participants from a Sicilian population provides a degree of homogeneity in terms of prenatal and postnatal environmental background. However, from a genetic perspective, it should be acknowledged that the contemporary Sicilian population has arisen from multiple historical admixture events, which may contribute to genetic heterogeneity within both the ASD and control groups. 

Another potential bias may arise from modest erythrocyte contamination (5–10%) or hemolysis, platelet contribution, and increased noise associated with low-abundance miRNAs. Although a direct analysis of cell composition was not performed, these factors represent plausible technical limitations. Consequently, the miRNA differences observed in this study should not be interpreted as evidence of a uniquely strong PBMC miRNA signature in ASD, but rather as a discovery-level dataset intended to guide targeted validation studies, reflecting a combination of disease-related variation and residual, un-modeled confounding effects. Also, because immune cell composition and inflammatory markers were not directly measured or modeled, the present study cannot disentangle disease-related regulatory effects from these sources of variability.

Similarly, comparison with the broader PBMC miRNA literature across psychiatric and immune-mediated disorders suggests that the magnitude and scale of differential expression observed here are atypical and may depend on cohort-specific immune characteristics. Without direct modeling of cytokine levels, inflammatory status, or immune cell composition, the present study cannot determine whether similar patterns would emerge in other neurodevelopmental or psychiatric conditions analyzed under identical conditions. Cross-disorder comparative studies are indeed essential to establish specificity.

Lastly, immune cell proportions and cytokine levels were not measured, precluding adjustment for immune activation state, which is known to strongly influence PBMC miRNA profiles. Moreover, no experimental validation of sequencing-based results was performed using orthogonal approaches, such as quantitative PCR, spike-in normalization, or independent replication cohorts. 

As a whole, these limitations prevent definitive attribution of the observed miRNA expression patterns to ASD-specific biology and underscore not only the exploratory and hypothesis-generating meaning of this study but also the need for further validation.

## 5. Conclusions

The preliminary data from this pilot study indicate the presence of altered miRNA regulatory patterns in ASD, adding support to the central role of inflammatory, autoimmune, and infectious mechanisms in this disorder, possibly mediated by miRNAs that regulate S100 family genes, neuronal migration, and synaptic communication. Nevertheless, these findings could not establish a predictive value or a diagnostic miRNA panel and, therefore, should be regarded as exploratory and hypothesis-generating. Confirmation in larger, independent cohorts with rigorous control of PBMC composition and orthogonal validation approaches are required before any conclusion regarding biomarker candidate or mechanistic causality can be drawn.

## Data Availability

The data are publicly available on ArrayExpress with access number E-MTAB-16206.

## Supplementary Materials

The following supporting information can be downloaded at: https://www.mdpi.com/article/10.3390/biomedicines14010217/s1. Table S1: Differentially Expressed miRNAs; Table S2: Diseases and Biofunctions; Table S3: miRNA TargetDegs Pathways Confidence; Table S4: Canonical Pathways_miRNA-Target; Table S5: Network.
